# Educational outcomes of emerging teaching methods in undergraduate nursing education: a systematic review and meta-analysis protocol

**DOI:** 10.1136/bmjopen-2025-101478

**Published:** 2025-05-21

**Authors:** Mohamad AlMekkawi, Mohammed Al Maqbali, Rouwida ElKhalil, Rasha Kadri Ibrahim, Aisha Aldawsari, Firas Qatouni, Moustafa Sherif, Suthan Pandarakutty, Sylivia Nalubega, Annie Rosita Arul Raj, Ciara Hughes

**Affiliations:** 1 Fatima College of Health Sciences, Abu Dhabi, UAE; 2 United Arab Emirates University, Al Ain, UAE; 3 School of Health Sciences, University of Ulster School of Nursing, Belfast, UK

**Keywords:** Virtual Reality, Augmented Reality, Nursing research

## Abstract

**Abstract:**

**Introduction:**

Undergraduate nursing education is essential in preparing competent and compassionate healthcare professionals capable of addressing the complex challenges in today’s healthcare landscape. This protocol proposes a systematic review of the educational outcomes of virtual/augmented reality, flipped classrooms, team-based learning and gamification compared with traditional or didactic methods in undergraduate nursing education.

**Methods and analysis:**

A systematic review protocol based on Preferred Reporting Items for Systematic Reviews and Meta-Analysis Protocol guidelines will be conducted. Experimental and observational studies published from 2014 through 2024 will be identified by searching the electronic databases PubMed, Scopus, Embase, Web of Science and CINAHL that compare emerging with traditional or didactic teaching methods among undergraduate nursing students. Two reviewers will independently assess titles and abstracts to identify relevant studies based on eligibility criteria. Two additional reviewers will extract data from full-text articles that meet these criteria, evaluate the risk of bias and assess the quality of the selected studies. The meta-analysis will include effect size, heterogeneity, subgroup analyses and publication bias tests.

**Ethics and dissemination:**

Ethics approval is not required. The publication will be in peer-reviewed journals and presented at national and international conferences.

**PROSPERO registration number:**

CRD42024618288.

**STRENGTHS AND LIMITATIONS OF THIS STUDY**

- This systematic review will be the first, of which we are aware, to examine the educational outcomes of various innovative teaching methods in undergraduate nursing education.
- The protocol is written following the Preferred Reporting Items for Systematic Review and Meta-Analyses Protocol guidelines.
- Limited by the exclusion of non-English papers.
- Challenges in meta-analyses are anticipated, given the heterogeneity of the study.

## Introduction

 Undergraduate nursing education is a foundation for developing competent and compassionate nursing professionals who are essential in tackling complex world healthcare challenges. These education programmes equip future nurses with comprehensive knowledge and the professional skills to deliver high-quality patient care.^1^ In an evolving, technology-driven healthcare landscape, educators must embrace innovative strategies to enhance nursing students’ critical thinking, encourage reflective practices and foster a sense of collective responsibility in preparation for their professional roles. Unfortunately, traditional teaching methods, particularly lecture-based or didactic, often fail to engage and inspire students.^2^ While these methods can effectively convey fundamental knowledge, they frequently do not foster the critical thinking skills essential for effective nursing practice.^3^ Such methods promote passive learning, which can impede the development of critical thinking abilities. Research indicates that passive learning techniques prioritise rote memorisation rather than fostering the analytical and evaluative skills necessary for enhancing students’ critical thinking and reasoning capabilities.^4 5^

Many teaching and learning methods have emerged in the last two decades to enhance nursing education and tackle existing challenges. Nursing educators are increasingly adopting student-centred and interactive methods that cater to diverse learning preferences. These emerging methods foster nursing students’ critical thinking, clinical judgement, active engagement and decision-making skills essential for specialised nursing care.^6^ In addition, they improve students’ comprehension and facilitate applying nursing theoretical models, ultimately preparing them for complex clinical settings.^7 8^ Additionally, research indicates that implementing technology-enhanced learning methods can significantly boost student engagement in higher education and prepare students for the increasingly digital nature of healthcare.^9 10^ Furthermore, scholars emphasise that cultivating active interactions between teachers and students fosters resilience and determination, which are crucial for motivating students and providing meaningful learning experiences that resonate with their interests.^11^

Four innovative teaching methodologies are transforming undergraduate nursing education by significantly enhancing student interactivity, engagement and active learning. These methodologies include virtual reality (VR), augmented reality (AR), the flipped classroom, team-based learning and gamification. Nursing education increasingly recognises VR and AR for their effectiveness in enhancing learning outcomes and clinical skills.^12^ VR uses technology to create an interactive three-dimensional environment, providing students with a sense of physical presence while disconnecting them from the real world.^13^ Conversely, AR preserves the student’s connection to their surroundings by overlaying virtual data, images and scenarios onto real-world visuals, allowing for the simultaneous experience of real and virtual elements.^14^ These immersive technologies offer nursing students realistic simulations and engaging experiences that enhance clinical skills training and boost self-efficacy, creating a safe environment for practice and skill development.^15^

The flipped classroom method represents a contemporary form of blended learning to enhance student engagement and learning outcomes. In this method, students complete assigned readings at home and engage in live problem-solving during class time.^16^ This asynchronous method optimises class time for student-centred synchronous learning.^17^ A systematic review examining the flipped classroom model for undergraduate nursing students indicated that participants experienced increased satisfaction and a greater sense of self-directed learning.^18^ A study involving Omani nursing students found that nearly 75% preferred the flipped classroom over traditional teaching methods, positively impacting their achievement and performance in anatomy and physiology courses.^19^

Moreover, integrating team-based learning (TBL) within nursing education has produced similarly positive outcomes. TBL is an active learning method encompassing preclass preparation, in-class collaboration and postclass development.^20^ This method enhances students’ interpersonal communication, critical thinking and clinical reasoning skills, which are essential for effective nursing practice.^21^,^23^ Furthermore, TBL’s intrinsic accountability and structured feedback mechanisms foster a supportive learning environment that can elevate student satisfaction.^24^ Additionally, gamification has emerged as a promising method for enhancing student motivation and engagement, although it is less commonly discussed in the context of nursing education.^25^ Gamification transforms learning activities into game-like experiences by incorporating elements such as charts, logos and scoring into non-game tasks to stimulate student engagement.^26^ By embedding these game-like features into the curriculum, educators can cultivate a more dynamic and engaging learning environment that boosts participation and fosters a sense of achievement among nursing students.^27^ A comparative summary of the key features and educational purposes of selected teaching methods is provided to enhance synthesis and clarity for readers (table 1).^12^,^27^

**Table 1:** Summary of selected teaching methods

Teaching method	Key features
Virtual reality	An immersive simulation that engages learners in interactive 3D environments using headsets. It enhances experiential learning by boosting psychomotor skills, critical thinking and confidence, particularly in complex tasks.
Augmented reality	A model that superimposes digital content, such as images and animations, onto the physical world using devices like smartphones and AR glasses. It fosters contextual understanding and engagement while keeping learners grounded in their real environments.
Flipped classroom	A model transforms traditional teaching by delivering instructional content, like video lectures, before class. This allows class time to focus on active, student-centred learning through discussions and problem-solving. It enhances students' engagement, deeper understanding and critical thinking.
Team-based learning	A collaborative learning model that encompasses individual preparation, readiness assurance testing and application-focused team activities. It promotes accountability, active participation and higher-order thinking while enhancing teamwork and communication skills essential in clinical practice.
Gamification	A model that integrates game elements into education to enhance motivation and engagement. It creates an interactive learning environment that encourages practice, improves retention and develops problem-solving skills in a fun and competitive way.

3D, three-dimensional.

Given the rapid advancements in educational technology and practices, evaluating the potential benefits of emerging teaching and learning methods in nursing education is vital. By comprehensively reviewing the emerging teaching methods compared with traditional approaches, we aim to identify strategies that best prepare nursing students for real-world challenges. This systematic review will critically evaluate the scientific evidence and provide valuable guidance to educators and decision-makers in developing more effective and engaging students’ learning experiences. Ultimately, these efforts will produce well-prepared nurses and enhance patient care. Therefore, this study will systematically review the educational outcomes of VR, AR, flipped classrooms, TBL and gamification compared with traditional or didactic methods in undergraduate nursing education.

The following research questions will guide this study:

1. What evidence exists on using VR, AR, flipped classrooms, TBL and gamification in undergraduate nursing education?
2. How do these emerging teaching methods impact students’ educational outcomes, specifically in skills acquisition, academic performance, student engagement, motivation and knowledge retention?

## Methods

This protocol adheres to items outlined under the Preferred Reporting Items for Systematic Reviews and Meta-Analysis (PRISMA) Protocol.^28^ This systematic review will follow the PRISMA guidelines.^29^ The review will comply with a protocol registered with the International Prospective Register of Systematic Reviews, PROSPERO (CRD42024618288).

### Patient and public involvement

None.

### Setting

This systematic review centres on educational institutions that provide baccalaureate nursing programmes, including universities, colleges and other accredited higher education facilities with undergraduate nursing students. The review explicitly explores studies conducted within formal educational settings, such as classrooms, simulation labs and clinical practice environments. Studies conducted in non-academic or graduate-level contexts will be excluded to ensure a focused examination of the undergraduate educational setting.

### Inclusion and exclusion criteria

Eligibility criteria will be based on the PICOS approach, which includes population, interventions, comparators, outcomes and study design. Eligible studies will be limited to those published between January 2014 and December 2024 to ensure relevance to recent innovations in teaching-learning strategies. The review will include studies published in English.

1. Population: We will consider studies involving undergraduate nursing students in recognised bachelor’s programmes. We will exclude studies involving non-nursing disciplines, graduate-level nursing education, or continuing education programmes.
2. Interventions: We will include studies examining VR or AR, flipped classrooms, TBL and gamification teaching methods compared with traditional or didactic methods. We will exclude studies that use different teaching methods from the review.
3. Comparators: The review will include all studies comparing the selected teaching methods with traditional or didactic methods. We will exclude studies using comparator teaching methods other than traditional or didactic ones.
4. Outcomes: Studies reporting on educational outcomes related to students’ clinical skills acquisition, academic performance, student engagement, satisfaction or knowledge retention will be considered for inclusion. We will exclude studies reporting other outcomes.
5. Study design: We will include empirical studies such as randomised controlled trials (RCTs), quasi-experimental studies, cohort studies, cross-sectional studies and case–control studies, which allow comparison of the selected teaching methods with traditional or didactic methods. Qualitative designs and non-empirical studies encompass grey literature, commentaries, editorials, book chapters, reports, review articles, case reports, case series, guidelines, websites and non-human studies will be excluded.

### Outcomes and outcome measures

In this systematic review, the following outcomes and outcome measures will be considered:

1. Students’ academic performance will be measured through standardised test scores, multiple-choice quizzes or pre- and postintervention assessments.^30^
2. Student skill acquisition will be measured using Objective Structured Clinical Examination scores, skills checklists or simulation performance rubrics.^31^
3. Student engagement will be measured through self-reported scales, such as the Intrinsic Motivation Inventory and the Student Engagement Questionnaire, or attendance rates.^32^
4. Student satisfaction and confidence will be measured through postintervention surveys, Likert-scale evaluations, structured interviews and focus groups.^33^
5. Knowledge retention: will be measured using post-tests or follow-up assessments.^34^

### Search strategy

We will thoroughly search PubMed, Scopus, Embase, Web of Science and CINAHL to identify studies that compare VR/AR, flipped classrooms, TBL and gamification with traditional or didactic teaching methods among undergraduate nursing students. We initially formulated the search in PubMed using MeSH terms (online supplemental file 1). An example of the PubMed search string is: (“nursing students” [MeSH Terms] OR “undergraduate nursing” [Title/Abstract]) AND (“virtual reality” [MeSH Terms] OR “augmented reality” [MeSH Terms] OR “flipped classroom” [Title/Abstract] OR “team-based learning” [MeSH Terms] OR “gamification” [Title/Abstract]) AND (“education”[MeSH Terms]). We will then make necessary adjustments to perform a similar search in the other databases for a systematic review (box 1). Boolean operators will be employed: (OR) will combine terms within the same category, while (AND or NOT) will combine or exclude terms from different categories. In addition, we will manually review the reference lists of relevant articles that meet the eligibility criteria to identify any additional suitable studies that may not have been uncovered through the database searches. Outcomes will not be included in the search criteria, as they could hinder the database’s ability to retrieve eligible studies. The specific outcomes may not be clearly articulated in the titles or abstracts.^35^

Box 1 **Systematic review search strategy**

Search strategy

- #1 Population (undergraduate nursing students)
- #2 Intervention (Virtual or Augmented Reality, Flipped Classrooms, Team-Based Learning and Gamification Teaching Methods)
- #3 #1 AND #2

### Study screening and selection

All articles retrieved from database searches will be imported into the Covidence Systematic Review software platform, where duplicates will be automatically removed. Two reviewers (SP and SN) will independently assess the titles and abstracts according to the specified eligibility criteria to identify relevant studies. These relevant studies will be reviewed to determine their inclusion in the final analysis. In cases of disagreement regarding the inclusion of a study, a third reviewer (MAMek) will make the final decision. After the initial screening, two independent researchers (FQ and AA) will evaluate the full texts of the retrieved studies for inclusion or exclusion. A third reviewer (MAMek) will resolve any disagreements and make the final decision. Reasons for excluding full-text papers that do not meet the inclusion criteria will be documented and reported.

### Data extraction

Two independent reviewers (FQ and AA) will conduct a pilot screening on 10% of the identified studies to ensure consistency and accuracy in the study selection process. This step aims to calibrate the reviewers and foster a shared understanding of the inclusion and exclusion criteria. Any discrepancies identified during the pilot screening will be discussed and resolved, with the criteria refined as necessary to enhance clarity.^36^ This approach aligns with established systematic review methodologies, ensuring methodological rigour and reliability in the final selection of studies.^29^

The reviewers (FQ and AA) will then start extracting data from full-text articles that meet the inclusion criteria using a Microsoft Excel 365 spreadsheet template created by the reviewers (online supplemental file 1). The extracted data will include descriptive characteristics that capture information about each study’s context, population, design and demographics, including the country, location, academic year, sample size, gender distribution and participant age. The second section will summarise educational interventions, including their design, the implemented learning strategies during classes, the preparatory methods and the comparative analyses of various groups. The final section will discuss the results of these studies, which include the educational outcomes measured, the instruments used, the results for the traditional and intervention groups and pertinent statistical data, such as means, SD and effect sizes.

### Data synthesis and statistical analysis

Fisher’s Z transformation of the correlation coefficient will be applied to assess the effect sizes of each teaching method compared with traditional or didactic methods. Since not all studies are expected to provide results as correlation coefficients, other effect size measures will be converted into correlation coefficients using established formulas.^37^ Missing data in effect size calculations will be addressed by contacting study authors for clarification or, where necessary, through mean imputation. Sensitivity analyses will evaluate the impact of these methods on overall findings.

Due to anticipated differences in study design, sample characteristics, interventions and outcome measures, a random effects model will be used to estimate the average effect size for each teaching method.^38^ This model will account for heterogeneity, assuming observed effects might vary across studies. Effect sizes will be evaluated according to Cohen’s criteria: r≤0.1 indicates a small effect, 0.3<r ≤ 0.5 points to a moderate effect, and r>0.5 signifies a large effect.^39^ 95% CI and 95% prediction intervals will be reported. A fixed-effects model is unsuitable because it relies on the assumption of a uniform actual effect size across all studies. This is unlikely due to the anticipated variability in educational settings and teaching strategy implementations. In contrast, the random-effects model effectively accommodates this variability, facilitating broader generalisability of the results.^38^

Heterogeneity among studies will be assessed using I² and Q statistics.^37^ I² values of 0–25% will indicate low heterogeneity, 25–50% moderate and 50–75% high.^40^ If high heterogeneity (I² > 50%) is observed, subgroup analyses or meta-regressions will be conducted to explore sources of variability. Subgroup analyses will focus on variables such as geographical region, publication year, sample size, and intervention type.

Publication bias will be examined with a funnel plot and tested statistically using Begg’s adjusted rank correlation test. A p-value of less than 0.05 will be considered statistically significant. All analyses will be conducted using R software (V.4.3.1, R Foundation for Statistical Computing) with the ‘meta’ package.^41^ Where bias is suspected, the trim-and-fill method will be applied to adjust effect size estimates. Sensitivity analyses will also exclude studies with a high risk of publication bias to evaluate the robustness of findings.

### Strength of the body of evidence

The studies featured in this review will be evaluated for evidence quality using the Grading of Recommendations Assessment, Development and Evaluation (GRADE) framework.^42^ This evaluation will use different GRADE domains to establish the overall strength of the evidence, which may be categorised as high, moderate, low or very low.^43^

### Risk of bias

For this meta-analysis, two reviewers (MAMaq and MS) will independently evaluate the risk of bias in eligible studies. The reviewers will first pilot 10% of the identified studies to ensure consistency and accuracy in the assessment. Any discrepancies identified during the pilot assessment will be discussed and resolved. The OHAT risk-of-bias tool,^44^ which adapts guidance from the Agency for Healthcare Research and Quality (AHRQ),^45^ will be used for this assessment (online supplemental file 1). The applicability of specific risk-of-bias questions depends on the study design, with different criteria used for RCTs, quasi-experimental studies and observational designs.^44^ Bias will be evaluated using 11 established questions that investigate (1) possible confounding factors, (2) consistency of exposure characterisation and (3) precision of outcome assessment. Each question will receive a rating of definitely low, probably low, probably high or definitely high-risk bias. After categorising individual responses, a three-tier system will assign an overall methodological quality rating.^46^ Studies in Tiers 1 and 3 represent high and low levels of methodological quality, respectively, while Tier 2 includes studies of intermediate quality. This tiered approach ensures a transparent and rigorous evaluation of the impact of educational interventions on learning outcomes, supporting reliable evidence synthesis.^46^

1. Tier 1: A study will be classified as Tier 1 if rated as ‘definitely low’ or ‘probably low’ risk of bias for key and most other applicable criteria.
2. Tier 2: A study will fall into Tier 2 if it does not meet the Tier 1 or Tier 3 criteria.
3. Tier 3: A study will be classified as Tier 3 if rated as ‘definitely high’ or ‘probably high’ risk of bias for key and most other applicable criteria.

For instance, an RCT deemed ‘definitely low’ risk in key areas—such as the randomisation process, confounding factors, exposure assessment and outcome measurement—would be classified as Tier 1, signifying high methodological quality. Conversely, a quasi-experimental study rated ‘probably low’ across most domains but raises concerns regarding exposure assessment or participant selection, which may be categorised as Tier 2, indicating intermediate quality. In contrast, a study assessed as ‘definitely high’ or ‘probably high’ risk of bias in critical aspects such as blinding or selective reporting would fall into Tier 3, reflecting low methodological quality.

## Discussion

This protocol examines the educational outcomes of various innovative teaching methods in undergraduate nursing education. Specifically, we will compare VR/AR, flipped classrooms, TBL and gamification against traditional or didactic methods. By evaluating these emerging teaching strategies, the study will provide valuable insights into their effects on students’ critical thinking, clinical skills, engagement, satisfaction and academic performance. Our findings will be significant for faculty members, nursing programmes and researchers as they consider adopting teaching methods that enhance students’ active learning and engagement in preparation for their future professional roles. For instance, nursing programmes should consider incorporating these approaches into their curricula if emerging teaching methodologies—such as team-based learning or flipped classrooms—yield significant improvements in student engagement, critical thinking or knowledge retention. Additionally, the findings may inform faculty development programmes by identifying pedagogical strategies that necessitate further training or support. Faculty can also leverage these insights to align their teaching methodologies with evidence-based educational outcomes, ultimately enhancing the overall quality and effectiveness of undergraduate nursing education. Additionally, our research may pave the way for future studies to explore or refine the application of these innovative teaching strategies.

Although this study is essential, we anticipate some limitations, such as variability in study designs, heterogeneity in outcome measures and potential publication bias. To address these concerns, we will implement a robust risk of bias assessment and employ appropriate statistical methods for data synthesis.

## Supplementary material

10.1136/bmjopen-2025-101478 online supplemental file 1
