# Supplementary material for: Educational outcomes of emerging teaching methods in undergraduate nursing education: a systematic review and meta-analysis protocol
Source: BMJ Open. 2025 May 21;15(5):e101478. doi: 10.1136/bmjopen-2025-101478 (PMC12097041; doi:10.1136/bmjopen-2025-101478)
Supplement: online supplemental file 1 [file bmjopen-15-5-s001.docx]

**The Educational Outcomes of Emerging Teaching Methods in Undergraduate Nursing Education: A Systematic Review and Meta-analysis Protocol**

**Supplementary file**

| **Content list** | **Page** |
| --- | --- |
| Supplementary 1A Search Strategies | 2-3 |
| Supplementary 1B Data Charting Instruments | 4 |
| Supplementary 1C RoB Checklist | 5 |

**Supplementary 1B Search Strategies**

|  | **1.1. Search for PubMed** | |
| --- | --- | --- |
| #1 | *Population Search* | "nurs* teach*"[Title/Abstract] OR "nurs* pedagog*"[Title/Abstract] OR "nurs* curric*"[Title/Abstract] OR "nurs* learn*"[Title/Abstract] OR "nurs* didact*"[Title/Abstract] OR "nurs* train*"[Title/Abstract] OR "nurs* simulat*"[Title/Abstract] OR "clinic* clerkship"[Title/Abstract] OR "Nursing Education Research"[MeSH Terms] OR "Clinical Clerkship"[MeSH Terms] OR "nurs* student*"[Title/Abstract] OR "undergraduat* nurs*"[Title/Abstract] OR "baccalaureate nurs*"[Title/Abstract] OR "nurs* research*"[Title/Abstract] OR "nurs* educat*"[Title/Abstract] OR "education, nursing, baccalaureate"[MeSH Terms] OR "Nursing Education Research"[MeSH Terms] OR "Nursing"[MeSH Terms] |
| #2 | *Intervention Search* | "Gamification"[MeSH Terms] OR "Artificial Intelligence"[MeSH Terms] OR "Computer Simulation"[MeSH Major Topic] OR "flipped class*"[Title/Abstract] OR "flipped learn*"[Title/Abstract] OR "augmented reality*"[Title/Abstract] OR "augmented reality*"[Title/Abstract] OR "Team-Based"[Title/Abstract] OR "team based*"[Title/Abstract] OR "virtual reality"[Title/Abstract] OR "virtual reality"[Title/Abstract] OR "gamification*"[Title/Abstract] OR "game based"[Title/Abstract] OR "game based"[Title/Abstract] OR "Artificial Intelligence"[Title/Abstract] OR "adaptive learning"[Title/Abstract] OR "adaptive learning"[Title/Abstract] OR "computer simulat*"[Title/Abstract] |
| #3 | *#1 AND #2* | ((("nurs* teach*"[Title/Abstract] OR "nurs* pedagog*"[Title/Abstract] OR "nurs* curric*"[Title/Abstract] OR "nurs* learn*"[Title/Abstract] OR "nurs* didact*"[Title/Abstract] OR "nurs* train*"[Title/Abstract] OR "nurs* simulat*"[Title/Abstract] OR "clinic* clerkship"[Title/Abstract] OR "Nursing Education Research"[MeSH Terms] OR "Clinical Clerkship"[MeSH Terms] OR "nurs* student*"[Title/Abstract] OR "undergraduat* nurs*"[Title/Abstract] OR "baccalaureate nurs*"[Title/Abstract] OR "nurs* research*"[Title/Abstract] OR "nurs* educat*"[Title/Abstract] OR "education, nursing, baccalaureate"[MeSH Terms] OR "Nursing Education Research"[MeSH Terms] OR "Nursing"[MeSH Terms]) AND ("Gamification"[MeSH Terms] OR "Artificial Intelligence"[MeSH Terms] OR "Computer Simulation"[MeSH Major Topic] OR "flipped class*"[Title/Abstract] OR "flipped learn*"[Title/Abstract] OR "augmented reality*"[Title/Abstract] OR "augmented reality*"[Title/Abstract] OR "Team-Based"[Title/Abstract] OR "team based*"[Title/Abstract] OR "virtual reality"[Title/Abstract] OR "virtual reality"[Title/Abstract] OR "gamification*"[Title/Abstract] OR "game based"[Title/Abstract] OR "game based"[Title/Abstract] OR "Artificial Intelligence"[Title/Abstract] OR "adaptive learning"[Title/Abstract] OR "adaptive learning"[Title/Abstract] OR "computer simulat*"[Title/Abstract])) NOT ("veterinar*"[Title/Abstract] OR "animal*"[Title/Abstract] OR "Animals"[MeSH Major Topic] OR "review"[Title] OR "poster"[Title] OR "conference"[Title])) AND (2014:2025[pdat]) |
|  | Result in documents  Final= 1,581 | |

**Note. The initial PubMed search was conducted on November 23, 2024

**Table A2.** Final Search Strategy

| **1.2. Search for Scopus** |
| --- |
|  |
| **1.3. Search for Embase** |
|  |
| **1.4. Search for Web of Science** |
|  |
| **1.5. Search for CINAHL** |
|  |

**Supplementary 1C Data Charting Instruments**

***1C I. Descriptive Characteristics of The Included Studies***

| ***#*** | **Study** | **(Authors/year)** | **Context** | | | | | | | **Population** | | | **Design** | | | | |
| --- | --- | --- | --- | --- | --- | --- | --- | --- | --- | --- | --- | --- | --- | --- | --- | --- | --- |
|  |  |  | **Country** | **Setting** | **Academic Year** | **Study Level** | **Course Name** | **Course Type** | **Duration** | **Total Sample** | **Gender (% Female)** | **Age Mean (SD)** | **Study Aim** | **Study Design** | **Randomization** | **Blinding** | **Sampling Method** |
|  |  |  |  |  |  |  |  |  |  |  |  |  |  |  |  |  |  |
|  |  |  |  |  |  |  |  |  |  |  |  |  |  |  |  |  |  |
|  |  |  |  |  |  |  |  |  |  |  |  |  |  |  |  |  |  |

***1C II. Summary of Studies on Educational Intervention***

| # | **(Authors/year)** | **Intervention Description** | | | **Intervention Design** | | | |
| --- | --- | --- | --- | --- | --- | --- | --- | --- |
|  |  | **Frequency** | **Pre-class preparatory strategies** | **Within-class active learning strategies** | **Comparative 1** | **Comparative 2** | **Comparative 3** | **Group  (Same, 2, 3…)** |
|  |  |  |  |  |  |  |  |  |
|  |  |  |  |  |  |  |  |  |
|  |  |  |  |  |  |  |  |  |

***1C III. Results of Studies on Educational Outcomes***

| # | **(Authors/year)** | **Educational Outcome measured** | **Instrument(s) used** | **Outcome Results** | **Traditional Group Outcome Information** | | | **Intervention Group Outcome Information** | | | |
| --- | --- | --- | --- | --- | --- | --- | --- | --- | --- | --- | --- |
|  |  |  |  |  | **Control No** | **Control mean** | **Control SD** | **Experimental No** | **Experimental mean** | **Experimental SD** | **Effect Size** |
|  |  |  |  |  |  |  |  |  |  |  |  |

**Supplementary 1C RoB Checklist**

| **Assessment Item** | **Article Rating** | **Comments/evidence** |
| --- | --- | --- |
| 1. Randomization of Exposure - Was the administered dose or exposure level adequately randomized? |  |  |
| 2. Allocation Concealment - Was allocation to study groups adequately concealed? |  |  |
| 3. Appropriate Comparison Groups - Did the selection of study participants result in appropriate comparison groups? |  |  |
| 4. Confounding and Modifying Variables - Did the study design or analysis account for important confounding variables? |  |  |
| 5. Identical Experimental Conditions - Were experimental conditions identical across study groups? |  |  |
| 6. Blinding During Study - Were research personnel and human subjects blinded to the study group during the study? |  |  |
| 7. Outcome Data Completeness - Were outcome data complete, without attrition or exclusion from analysis? |  |  |
| 8. Exposure Characterization - Can we be confident in the exposure characterization? |  |  |
| 9. Outcome Assessment Blinding - Can we be confident in the outcome assessment? |  |  |
| 10. Selective Reporting - Was there selective reporting of results? |  |  |
| 11. Other Bias Threats - Any other potential threats to internal validity. |  |  |
| **Final score** |  | |
