# Supplementary material for: Educational outcomes of emerging teaching methods in undergraduate nursing education: a systematic review and meta-analysis protocol
Source: BMJ Open. 2025 May 21;15(5):e101478. doi: 10.1136/bmjopen-2025-101478 (PMC12097041; doi:10.1136/bmjopen-2025-101478)
Supplement: Supplementary file 2 [file bmjopen-2025-101478.reviewer_comments.pdf]

PEER REVIEW HISTORY

BMJ Open publishes all reviews undertaken for accepted manuscripts. Reviewers are asked to complete a checklist review form and are provided with free text boxes to elaborate on their assessment. These free text comments are reproduced below.

ARTICLE DETAILS

Title (Provisional)

The Educational Outcomes of Emerging Teaching Methods in Undergraduate Nursing Education: A Systematic Review and Meta-analysis Protocol

Authors

AlMekkawi , Mohamad; Al Maqbali, Mohammed; ElKhalil, Rouwida; Ibrahim , Rasha Kadri; Aldawsari , Aisha; Qatouni , Firas; Sherif, Moustafa; Pandarakutty , Suthan; Nalubega , Sylivia; Arul Raj, Annie Rosita; Hughes, Ciara

---

VERSION 1 - REVIEW

**Reviewer**	**1**
**Name**	**Magi, Camilla Elena**
**Affiliation**	**University of Florence**
**Date**	**23-Apr-2025**
**COI**	**None**

Thank you for the opportunity to review this manuscript. I appreciate the chance to contribute to the evaluation of such a relevant and timely topic.

The subject matter addressed is compelling and adds meaningful novelty to the literature on innovative teaching methods in undergraduate nursing education. The manuscript demonstrates solid potential to provide valuable contributions to both research and practice.
However, I have identified several areas where the manuscript could benefit from refinement.

Below are detailed suggestions organized by section:

- **Abstract**
  - Use more tentative language when presenting expected outcomes (e.g., "may provide insights" instead of "will provide").
- **Introduction**
  - Consider dividing dense paragraphs (especially lines 47–80) to enhance clarity and flow.
  - A brief comparative table summarizing the key features of the selected teaching methods (VR, AR, flipped classroom, TBL, gamification) would improve readability and synthesis.
- **Objectives and Research Questions**

    - Well-defined and consistent with the PICO framework — no revisions needed.
- **Methods**
    - *Inclusion Criteria*: Clarify whether the literature search will be updated to include studies from 2025, given the review period extends until December 2025.
    - *Search Strategy*: Although well-detailed in the appendix, consider including a brief example or summary of the search string in the main text for transparency.
    - *Statistical Analysis*: Briefly justify the choice not to use a fixed-effects model, to strengthen methodological transparency.
- **Risk of Bias Assessment**
    - Adding an example (real or hypothetical) of how studies are classified into Tier 1, 2, or 3 would increase the clarity of your quality assessment approach.
- **Discussion**
    - Strengthen this section by commenting on how the results could inform practical recommendations for educators and curriculum developers.
- **Ethics, Funding, and Declarations**
    - Clearly reported — no modifications needed.

In summary, the manuscript is promising and relevant. Addressing the points above will further improve its clarity, rigor, and practical impact.

I look forward to reviewing the revised version and seeing its contribution to the field.

**Reviewer**	**2**
**Name**	**Mendez, Kyra**
**Affiliation**	**National Cancer Institute**
**Date**	**30-Apr-2025**
**COI**	**None**

Thank you for the opportunity to review this protocol. It is a well-written and well-designed protocol, and this study will be very relevant to the nursing education field. Some revisions suggested:

There is a discrepancy in the time period for study inclusion – in the abstract (lines 12-13), it indicates studies published from 2014 to 2025, but in the methods (line 142), it indicates studies published between 2015 and 2024. Please clarify the time period and ensure consistency throughout protocol.

The authors state, “The quality of evidence from the studies included in this review will be assessed using the Grading of Recommendations Assessment, Development, and Evaluation (GRADE) framework 37.” However, reference 37 is the COSMIN guideline; please clarify.

The reference (38) for the three-tier system for methodological quality rating does not appear to be correct. Reference 38 uses a four-tier system (See Table 2 Confidence Ratings in

Reference 38). Upon Googling, it appears the 3-tier system may be coming from doi:10.1177/1091581818754330. Please clarify.

Some of the sentences are very close to (or exactly the same as) the original sources (e.g., lines 249-251 "Individual risk-of-bias questions are designated as applicable only to specific types of study designs" is identical to Reference 38). Please ensure quotations are used for content that comes directly from sources or summarize.

VERSION 1 - AUTHOR RESPONSE

Reviewer 1 Comments	Author Response
**Abstract**<br>Use more tentative language when presenting expected outcomes (e.g., "may provide insights" instead of "will provide").	Thank you for your helpful comment. The expected results section in the abstract was removed as per the journal Instructions for Authors.
**Introduction**<br>• Consider dividing dense paragraphs (especially lines 47–80) to enhance clarity and flow.<br>• A brief comparative table summarizing the key features of the selected teaching methods (VR, AR, flipped classroom, TBL, gamification) would improve readability and synthesis.	Thank you for your helpful comments.<br><br>The long paragraph is separated to enhance clarity (lines 49-50).<br><br>In the Introduction section, we added a comparative table summarizing the selected teaching methods' key features and educational purposes (lines 94-99).
**Objectives and Research Questions**<br>Well-defined and consistent with the PICO framework — no revisions needed.	Thank you.
**Methods**<br><br>Inclusion Criteria: Clarify whether the literature search will be updated to include studies from 2025, given the review period extends until December 2025.<br><br>Search Strategy: Although well-detailed in the appendix, consider including a brief example or summary of the search string in the main text for transparency.<br><br>Statistical Analysis: Briefly justify the choice not to use a fixed-effects model, to strengthen methodological transparency.	Thank you for the valuable feedback.<br><br>The review will include studies published between January 2014 and December 2024 to align with our planned timeline for analysis and reporting. We have updated the manuscript for clarity and consistency (lines 119, 139-40).<br><br>We have revised the manuscript to include a concise example of the PubMed search string in the main text (lines 178-81) while retaining the detailed strategy in Supplementary 1.<br><br>We have revised the "Data Synthesis and Statistical Analysis" section to briefly justify our choice not to use a fixed-effects model (lines 135-39).
**Risk of Bias Assessment**	

Adding an example (real or hypothetical) of how studies are classified into Tier 1, 2, or 3 would increase the clarity of your quality assessment approach.	Thank you for your suggestion. We added a hypothetical example that illustrates how studies are classified into Tier 1, 2, or 3 based on their methodological characteristics using the OHAT tool (lines 278-85).
**Discussion**<br>Strengthen this section by commenting on how the results could inform practical recommendations for educators and curriculum developers.	Thank you for your helpful comment. We have revised the Discussion section to include a paragraph outlining how the anticipated results of our review can inform practical recommendations for educators (lines 295-302).
**Ethics, Funding, and Declarations**<br>Clearly reported — no modifications needed.	Thank you.
**Reviewer 2 Comments**	**Author Response**
1. There is a discrepancy in the time period for study inclusion – in the abstract (lines 12-13), it indicates studies published from 2014 to 2025, but in the methods (line 142), it indicates studies published between 2015 and 2024. Please clarify the time period and ensure consistency throughout protocol.	Thank you for highlighting the discrepancy regarding the study inclusion period. We can confirm that the inclusion timeline is from 2014 to 2024. The reference to 2025 in the abstract was an unintentional mistake, and we have revised the text to maintain consistency throughout the manuscript (lines 12, 119, 139-40).
2. The authors state, "The quality of evidence from the studies included in this review will be assessed using the Grading of Recommendations Assessment, Development, and Evaluation (GRADE) framework 37." However, reference 37 is the COSMIN guideline; please clarify.	Thank you for pointing out this inconsistency. We acknowledge that the citation for the COSMIN guideline was inaccurate. We have revised the references to include two relevant sources for the GRADE approach (Guyatt et al., 2008, and Prasad, 2024, lines 253-56).
3. The reference (38) for the three-tier system for methodological quality rating does not appear to be correct. Reference 38 uses a four-tier system (See Table 2 Confidence Ratings in Reference 38). Upon Googling, it appears the 3-tier system may be coming from doi:10.1177/1091581818754330. Please clarify.	Thank you for your careful review. You are correct; reference 38 (Rooney et al., 2014) outlines a four-tier confidence rating system, not the three-tier methodological quality rating system described in our protocol. The three-tier system we reference is adapted from Wikoff et al. (2018). We have corrected the citation in the manuscript (line 269) and updated the reference list accordingly.
4. Some of the sentences are very close to (or exactly the same as) the original sources (e.g., lines 249-251 "Individual risk-of-bias questions are designated as applicable only to specific types of study designs" is identical to Reference 38). Please ensure quotations are used for content that comes directly from sources or summarize.	Thank you for pointing this out. We acknowledge that some sentences were too closely aligned with the source language. We have revised the relevant sections and paraphrased the content appropriately (lines 233-34; 263-68).
